# Decreased number of mast cells infiltrating into needle biopsy specimens leads to a better prognosis of prostate cancer

**DOI:** 10.1038/sj.bjc.6603962

**Published:** 2007-09-11

**Authors:** N Nonomura, H Takayama, K Nishimura, D Oka, Y Nakai, M Shiba, A Tsujimura, M Nakayama, K Aozasa, A Okuyama

**Affiliations:** 1 Department of Urology, Osaka University Graduate School of Medicine, Osaka, Japan; 2 Department of Pathology, Osaka University Graduate School of Medicine, Osaka, Japan

**Keywords:** biopsy, immunostaining, mast cells, prognostic factor, prostate cancer

## Abstract

Mast cell infiltration is often observed around human tumours. Inflammatory cells such as macrophages, neutrophils and mast cells infiltrating around tumours are known to contribute to tumour growth; however, the clinical significance of mast cell invasion in prostate cancer (PCa) has not been investigated. Mast cell infiltration was evaluated in 104 patients (age range, 45–88 years; median, 72 years), who underwent needle biopsy of the prostate and were confirmed to have PCa. Needle biopsy specimens of prostate were sliced into 5-*μ*m-thick sections and immunostained for mast cells with monoclonal antibody against mast cell-specific tryptase. Mast cells were counted systematically under a microscope (× 400 magnification), and the relations between mast cell numbers and clinicopathologic findings were evaluated. The mast cell count was evaluated for prognostic value by multivariate analysis. Mast cells were immunostained around the cancer foci. The median number of mast cells in each case was 16. The mast cell count was higher around cancer foci in patients with higher Gleason scores than in those with low Gleason scores. The mast cell number correlated well with clinical stage (*P*<0.001). Prostate-specific antigen-free survival of patients with higher mast cell counts was better than that in patients with lower mast cell counts (*P*<0.001). Multivariate analysis revealed that mast cell count was a significant prognostic factor (*P*<0.005). The number of mast cells infiltrating around cancer foci in prostate biopsy specimens can be a significant prognostic factor of PCa.

Prostate cancer (PCa) is one of the most common malignancies and is now the second leading cause of cancer-related death in men in the United States (Parker *et al*, 1996; Parkin *et al*, 2002). Several prognostic factors related to the clinicopathologic features have been described. Among them, serum prostate-specific antigen (PSA) level, T stage and Gleason score are considered very important prognostic factors (Partin *et al*, 1997; Pound *et al*, 1997; Kattan *et al*, 1998; Hull *et al*, 2002). However, only a few factors that represent the host response which is composed of various inflammatory cells have been reported as prognostic factors for PCa so far (Irani *et al*, 1999; Wise *et al*, 2000; Schulz *et al*, 2007).

Tumour cells are surrounded by inflammatory cells such as macrophages, lymphocytes, neutrophils and mast cells. Inflammatory cell infiltrates, particularly macrophages, may contribute to tumour progression by producing angiogenic factors (Gruber *et al*, 1995; Jenkins *et al*, 1995). The role of mast cells in tumour growth has been studied extensively in experimental tumours (Fisher and Fisher, 1965; Tanooka *et al*, 1982; Burtin *et al*, 1985), however, the significance of mast cell infiltration around human tumours has not been well studied, even though accumulation of mast cells around tumours was first reported a decade ago (Dimitriadou and Koutsilieris, 1997). Most studies have shown that mast cells prevent tumour growth (Farram and Nelson, 1980; Henderson *et al*, 1981; Ghiara *et al*, 1985; Benyon *et al*, 1991). In contrast, in a heterocyclic amine-induced rat PCa model, Nakai *et al* (2007) reported increased infiltration of stromal mast cell in the ventral prostate, with increased prostatic epithelial proliferation, suggesting that mast cells may have beneficial roles in progression of PCa (Nakai *et al*, 2007). The significance of mast cell accumulation around PCa has not been investigated extensively (Gupta, 1970; Sari *et al*, 1999; Aydin *et al*, 2002). In the present study, the viability of mast cell accumulation around PCa as a prognostic factor was examined.

## MATERIALS AND METHODS

### Patients

One hundred and four patients diagnosed with local PCa by transrectal needle biopsy at Osaka University Hospital between 1997 and 2000 were selected for the present study. Written informed consent form approved by our institute was obtained. The ages of the patients ranged from 45 to 88 years (median, 72 years). A diagnosis of PCa was made by histologic examination of the biopsy specimens. Clinical stage was defined according to the American staging system (Sobin and Wittekind, 1997) through digital rectal examination (DRE), transrectal ultrasonography, chest X-ray, computed tomography, magnetic resonance imaging and bone scintigram. Serum PSA levels measured by immunoenzymatic assay ranged from 4.3 to 316.8 ng ml^−1^ (median, 16.9 ng ml^−1^). Clinical stage was distributed as follows: 20 cases (19.2%) in stage T1, 46 cases (44.2%) in stage T2, 36 cases (34.6%) in stage T3 and two cases (1.9%) in T4 (Table 1). Biopsy specimens were fixed in 10% neutral buffered formalin and routinely processed for paraffin embedding. Serial 5-*μ*m-thick sections were cut, stained with haematoxylin and reviewed by one pathologist to determine the Gleason score (Gleason and Mellinger, 1974).

Seventy-five patients underwent radical prostatectomy and remaining 29 received irradiation therapy as initial therapy. No patients received brachytherapy. After initial therapy, patients were followed up with periodic evaluations of DRE, serum PSA and imaging findings. Progression of PCa was defined as an elevated PSA at three consecutive measurements (PSA failure), the appearance of new lesion, reappearance of any lesion that had disappeared or clear worsening of non-measurable disease.

### Immunohistochemical analyses

Mast cells were stained immunohistochemically with a monoclonal antibody against tryptase specific to mast cells (TransGenic Inc., Kobe, Japan). Immunohistochemistry of paraffin sections was carried out with an LSAB kit (Dako, Glostrup, Denmark). For systematic counting, three ocular measuring fields, each with a real area of 0.06175 mm^2^, were randomly chosen under a microscope at a power of × 400 within a section. If the cancer foci were too small to obtain three independent fields, mast cells were counted in only one or two fields.

### Statistical analyses

Statistical analyses were performed with StatView software (SAS Institute Inc., Cary, NC, USA). Correlation between mast cell count by immunohistochemistry and clinicopathologic parameters was evaluated with the *χ*^2^ test (Table 2). The follow-up period for survivors measured from the date of start of therapy, ranged from 2.7 to 181.5 months (mean, 54.5 months). Progression-free survival rate was calculated by the Kaplan–Meier method, and differences in survival curves were estimated with the Log-rank test (Figures 2 and 3). Independent prognostic factors were analysed by Cox proportional hazards regression model in a stepwise manner. *P*<0.05 was considered statistically significant.

## RESULTS

### Mast cell infiltration of PCa

**Figure 1:**
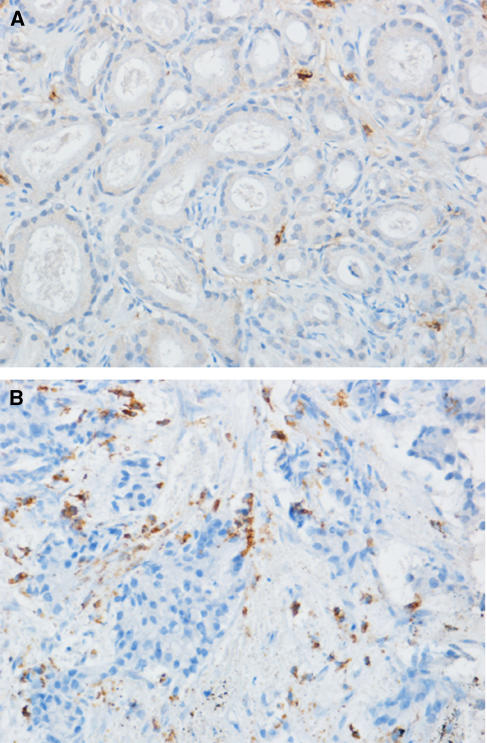
Immunostaining of mast cells with monoclonal antibody against mast cell-specific tryptase in prostate biopsy specimens. (**A**) A case with high-level mast cell infiltration. (**B**) A case with low-level mast cell infiltration. Bar, 20 *μ*m.

Matriptase-positive cells (mast cells) were observed only around the cancer foci (Figure 1). The median number of mast cells infiltrating into the peritumoral area was 16 (range, 5–70). Of the 104 cases, 52 cases had a mast cell count higher than 16 (designated as ‘high MC group’), and the remaining 52 patients had a mast cell count lower than 16 (low MC group).

### Correlations between mast cell count and various clinicopathologic factors

The correlations between mast cell count and various clinicopathologic factors are shown in Table 2. Statistical significance was calculated by *χ*^2^ test. The mast cell count correlated positively with clinical T (*P*<0.001) and with Gleason score (*P*<0.05, Gleason score ⩽6 *vs* 7; *P*<0.001, Gleason score ⩽6 *vs* ⩾8; *P*<0.05, Gleason score 7 *vs* ⩾8). Patients with PSA failure had significantly higher mast cell counts than those with no PSA failure (*P*<0.001). However, PSA level did not correlate significantly with mast cell count.

### Recurrence-free survival analysis of patients treated by radical prostatectomy or irradiation therapy as initial therapies

**Figure 2:**
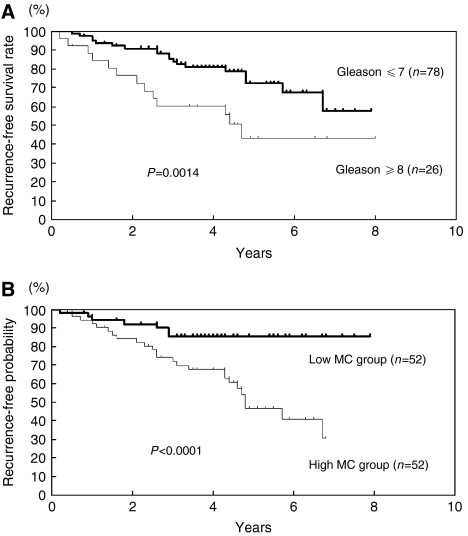
Progression-free survival of PCa patients stratified by Gleason score (GS) or mast cell infiltration. (**A**) Progression-free survival of PCa patients with high and low Gleason scores. (**B**) Progression-free survival of PCa patients in high and low mast cell (MC) counts. High Gleason score, GS⩾8; low Gleason score, GS⩽7. High MC group, >16; low MC group, ⩽16.

Progression-free survival rates of 104 patients who underwent radical prostatectomy or irradiation therapy as initial therapies were evaluated. The follow-up period ranged from 2.0 to 96.0 months (mean, 46.8 months). The disease-free survival time ranged from 2.0 to 84.2 months (mean, 32.9 months). Progression-free survival of the patients with low Gleason score (<8) was significantly better than those with high Gleason score (⩾8), as expected (*P*<0.005) (Figure 2A). The median progression-free survival time of the patients with high Gleason scores was 56 months. But, the progression-free survival rate of the patients with low Gleason score did not reach 50% during the observation period. The significance of mast cell infiltration for progression-free survival was also analysed. Progression-free survival of the low MC group was significantly better than that of the high MC group (*P*<0.0001) (Figure 2B). The median progression-free survival time of the high MC group was 57 months. However, the median progression-free survival time of the low MC group did not reach 50% during the observation period.

### Recurrence-free survival of patients treated with androgen deprivation therapy

**Figure 3:**
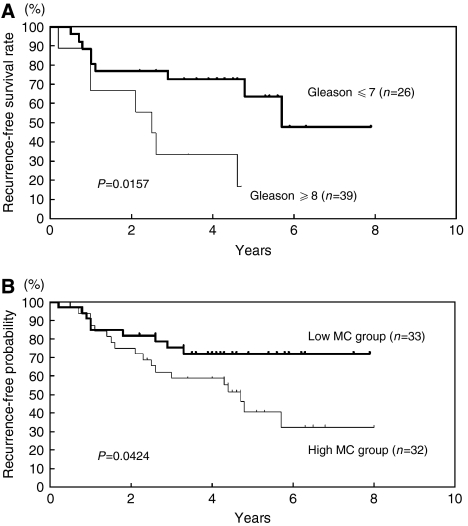
Progression-free survival of PCa patients treated with androgen deprivation therapy stratified by Gleason score or mast cell infiltration. (**A**) Progression-free survival of PCa patients with high and low Gleason scores. (**B**) Progression-free survival of PCa patients with high and low mast cell (MC) counts. High Gleason score, GS⩾8; low Gleason score, GS⩽7. High MC group, >16, low MC group, ⩽16.

Androgen deprivation therapy is a commonly used therapy for PCa in Japan. Therefore, we examined progression-free survival of patients who received only androgen deprivation therapy. The progression-free survival in patients with low Gleason score or patients in the low MC group was better than that of patients with high Gleason score or in the high MC group (*P*<0.05) (Figure 3A and B). The median progression-free survival time of the patients with high and low Gleason scores were 30 and 68 months, respectively. The median progression-free survival of the high MC group was 57 months, but the progression-free survival rate of the low MC group did not reach 50% during the observation period.

### Cox multivariate analysis for prognostic factors

Results of Cox multivariate analysis are shown in Table 3. Mast cell infiltration (mast cell count >16), Gleason score (>7) and extraprostatic extension (positive) were significant factors for progression-free survival. However, mast cell infiltration had the lowest *P*-value and the highest hazard ratio (2.726).

## DISCUSSION

It has been long recognized that inflammatory cell infiltration is often seen around many types of tumours (Dimitriadou and Koutsilieris, 1997). Most attention focused on the role of infiltration lymphoid cells such as tumour-infiltrating lymphocytes, which represent the host immune reaction against the tumour (Romero *et al*, 1998; Molldrem *et al*, 2000; Zhang *et al*, 2003). There have been a number of reports regarding mast cell infiltration around a variety of human and experimentally induced tumours (Ueda *et al*, 1988; Dimitriadou and Koutsilieris, 1997; Takanami *et al*, 2000; Ribatti *et al*, 2004; Tuna *et al*, 2006).

In general, peripheral distribution of mast cells around an experimentally induced tumour has been recognized as playing a protective role against the tumours (Farram and Nelson, 1980; Tanooka *et al*, 1982; Burtin *et al*, 1985). In these tumours, mast cells and their degranulation products (histamine, serotonin and heparin) have been reported to be involved in tumour inhibition (Tharp *et al*, 1989; Benyon *et al*, 1991). It has been reported that the incidence of metastases as well as the appearance of tumours correlates inversely with tissue histamine level and mast cell count (Burtin *et al*, 1985). Interestingly, isolated mast cells were shown to inactivate tumour cells selectively *in vitro* (Henderson *et al*, 1981). These findings suggest that mast cells may play a role in inhibiting tumour metastases and suppressing growth of primary tumours. The protective roles of mast cell infiltration have been reported in soft tissue sarcomas in both human and animal models (Ueda *et al*, 1988).

In contrast to the findings in experimentally induced malignancies, some studies have suggested promote that mast cells tumour progression. For instance, mast cell infiltration correlates well with tumour angiogenesis and metastases in gastric cancer (Yano *et al*, 1999), colorectal cancer (Lachter *et al*, 1995), pulmonary adenocarcinoma (Takanami *et al*, 2000) and renal cell carcinoma (Tuna *et al*, 2006). In PCas, mast cell infiltration correlates with poor prognosis, suggesting that mast cells may promote tumour progression. Interestingly, in our series of studies, a low mast cell count was associated with longer recurrence-free survival of the patients receiving hormonal therapies. In some immunohistochemical studies, significant associations between mast cell number and microvessel density were reported (Takanami *et al*, 2000; Tuna *et al*, 2006). Mast cells are known to produce several mediators of angiogenesis, including fibroblast growth factor (FGF)-2, vascular endothelial cell growth factor (VEGF), transforming growth factor (TGF)-*β* and interleukin (IL)-8 (reviewed by Ribatti *et al*, 2004). These factors are all involved both in normal as well as tumour-associated angiogenesis. In addition to these angiogenic factors, mast cells also produce proteolytic enzymes such as matrix metalloproteinase (MMP)-2 and -9, and tryptase (Fang *et al*, 1999; Vincent *et al*, 2000; Di Girolamo *et al*, 2006). These factors are known to promote invasion of cancer cells into interstitial stromal tissue. Moreover, tryptase is one of the proteolytic enzymes produced specifically by mast cells. In the present study, we stained mast cells in prostate biopsy specimens with antibody against mast cell-specific tryptase. Mast cell-specific tryptase may also promote cancer cell invasion (Stack and Johnson, 1994; Blair *et al*, 1997). Because microvessels are usually very sparse in prostate biopsy specimens, the correlation between microvessels and mast cell infiltration was not examined in the present study.

Gupta (1970) first reported the presence of mast cells around PCas. In our study, we observed mast cell aggregation at the periphery of PCas, similar to previous reports (Sari *et al*, 1999; Aydin *et al*, 2002). However, this is the first report that mast cell infiltration around prostate tumours is a significant prognostic factor for PCa.

## Figures and Tables

**Table 1:** Patient characteristics

	**Median (range)**
Age (years)	72 (45–88)
Prostate-specific antigen (ng/ml)	16.9 (4.3–316.8)
	
*Gleason score*	
⩽6	49 (44.2%)
7	29 (27.9%)
⩾8	26 (25.0%)
	
*Clinical T stage*	
T⩽2	66 (63.4%)
T⩾3	38 (36.5%)
	
Total	104 (100%)

**Table 2:** Correlation between mast cell infiltration and clinicopathologic factors

**Clinicopathologic factors**	**Number of patients (%)**	**Mast cell count Mean (s.e.)^a^**	***P*-value**
*Age (years)*			
<70	51 (49%)	20.35 (13.25)	NS
⩾70	53 (51%)	20.11 (11.83)	
			
*PSA*^b^ *(ng/ml)*			
<20	54 (51.9%)	18.59 (10.08)	NS
⩾20	50 (48.1%)	21.74 (14.29)	
			
*Gleason score (GS)*			
GS⩽6	49 (44.2%)	15.80 (8.03)	<0.05^c^
GS=7	29 (27.9%)	20.98 (13.94)	<0.001^d^
GS⩾8	26 (25.0%)	27.74 (14.25)	<0.05^e^
			
*Clinical T stage*			
⩽T2	66 (63.4%)	17.30 (8.26)	<0.001
⩾T3	38 (36.5%)	25.10 (16.39)	
			
*PSA failure*			
+	30 (28.8%)	27.56 (15.70)	<0.001
−	74 (71.2%)	16.67 (8.69)	

NS, not significant.

a Standard error.

b Prostate-specific antigen.

c GS⩽6 *vs* GS=7.

d GS⩽6 *vs* GS⩾8.

e GS=7 *vs* GS⩾8.

**Table 3:** Prognostic factors by Cox regression analysis

**Clinicopathologic factors**	**Hazard ratio**	**95% confidence interval**	***P*-value**
Age (continuous variable)	0.979	0.609–1.575	0.9312
PSA level at biopsy (continuous variable)	0.999	0.997–1.000	0.526
Gleason score >7	1.635	1.000–2.671	0.0498
DRE positive	0.814	0.385–1.721	0.5907
Extraprostatic extension (+)	2.616	1.389–4.925	0.0029
Lymph node metastasis (+)	0.978	0.200–4.778	0.9778
Mast cell count >16	2.726	1.476–5.034	0.0014

DRE=digital rectal examination; PSA=prostate-specific antigen.
